# High-throughput Proteomics Identifies THEMIS2 as Independent Biomarker of Treatment-free Survival in Untreated CLL

**DOI:** 10.1097/HS9.0000000000000951

**Published:** 2023-09-15

**Authors:** Paul J. Hengeveld, P. Martijn Kolijn, Jeroen A.A. Demmers, Wouter Doff, Julie M.N. Dubois, Melissa Rijken, Jorn L.J.C. Assmann, Lina van der Straten, Henk Jan Boiten, Kirsten J. Gussinklo, Peter J.M. Valk, Laura M. Faber, Peter E. Westerweel, Arnon P. Kater, Mark-David Levin, Anton W. Langerak

**Affiliations:** 1 Department of Immunology, Erasmus MC, Rotterdam, the Netherlands; 2 Department of Internal Medicine, Albert Schweitzer Hospital, Dordrecht, the Netherlands; 3 Proteomics Center, Erasmus MC, Rotterdam, the Netherlands; 4 Department of Hematology and Experimental Immunology, Cancer Center Amsterdam, Amsterdam University Medical Centers, University of Amsterdam, the Netherlands; 5 Department of Hematology, Erasmus MC Cancer Institute, University Medical Center Rotterdam, the Netherlands; 6 Department of Hematology, Red Cross Hospital, Beverwijk, the Netherlands

## Abstract

It remains challenging in chronic lymphocytic leukemia (CLL) to distinguish between patients with favorable and unfavorable time-to-first treatment (TTFT). Additionally, the downstream protein correlates of well-known molecular features of CLL are not always clear. To address this, we selected 40 CLL patients with TTFT ≤24 months and compared their B cell intracellular protein expression with 40 age- and sex-matched CLL patients with TTFT >24 months using mass spectrometry. In total, 3268 proteins were quantified in the cohort. Immunoglobulin heavy-chain variable (IGHV) mutational status and trisomy 12 were most impactful on the CLL proteome. Comparing cases to controls, 5 proteins were significantly upregulated, whereas 3 proteins were significantly downregulated. Of these, only THEMIS2, a signaling protein acting downstream of the B cell receptor, was significantly associated with TTFT, independently of IGHV and *TP53* mutational status (hazard ratio, 2.49 [95% confidence interval, 1.62-3.84]; *P* < 0.001). This association was validated on the mRNA and protein level by quantitative polymerase chain reaction and ELISA, respectively. Analysis of 2 independently generated RNA sequencing and mass spectrometry datasets confirmed the association between THEMIS2 expression and clinical outcome. In conclusion, we present a comprehensive characterization of the proteome of untreated CLL and identify THEMIS2 expression as a putative biomarker of TTFT.

## Graphical abstract

**Figure:**
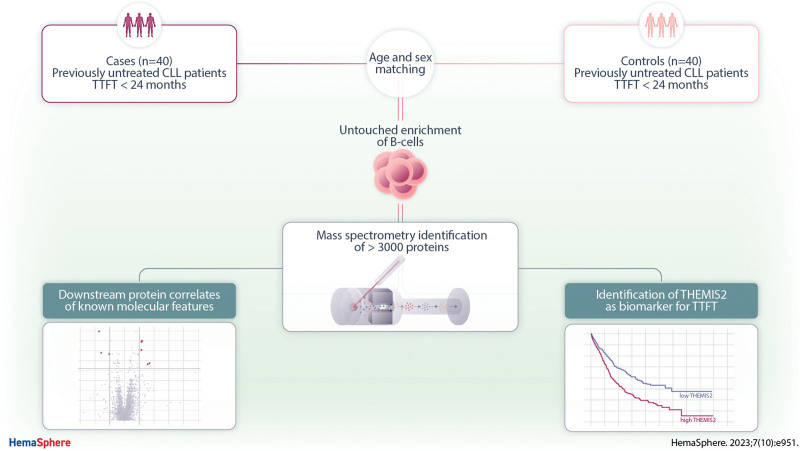

## INTRODUCTION

The interval from diagnosis to the development of symptomatic disease necessitating treatment can vary considerably between patients with chronic lymphocytic leukemia (CLL). Although some patients may have stable disease for years, others experience rapid progression and require treatment within months following diagnosis. Heterogeneity in disease biology underpins this clinical variability. Indeed, the molecular landscape of CLL is characterized by numerous recurrently encountered aberrancies, rather than having a common single driver.^1,2^

An important source of variability in CLL is the immunoglobulin heavy-chain (IGH) variable (IGHV) mutational status. According to the burden of somatic hypermutation (SHM) present in the *IGHV* gene, CLL is classified as unmutated or mutated CLL (U-CLL or M-CLL), likely reflecting pre and postgerminal center ancestry. M-CLL is associated with a considerably longer time-to-first treatment (TTFT) compared with U-CLL.^3,4^ Other important recurrent molecular drivers are numerous cytogenetic anomalies, including trisomy 12, deletions of chromosome 13q14, 11q22, or 17p13, genomic complexity, and single-gene lesions, most notably affecting the *TP53* gene.^5–7^

Although characterization of some of these features is integrated in routine CLL care, it remains challenging to recognize CLL patients with unfavorable TTFT at an early disease stage, especially in the setting of M-CLL with intact *TP53*. In addition, the pathophysiological downstream effects of several well-known molecular features of CLL are not completely clear. To address these issues, we performed large-scale characterization of global intracellular protein expression by mass spectrometry in a cohort of previously untreated CLL patients, to identify downstream protein correlates of molecular features of CLL and find putative biomarkers associated with inferior TTFT.

## MATERIALS AND METHODS

### Patient samples

Patient samples were obtained from the biobank for B-cell malignancies, coordinated by the Academic Medical Center in Amsterdam, the Netherlands. Patients provided written informed consent before enrollment and inclusion of samples in the biobank. Ethics approval was provided by the Amsterdam University Medical Centers medical ethical and biobank committees (METC 2013_159), in accordance with the Declaration of Helsinki. Selection criteria for the mass spectrometry and validation cohorts included the following: (1) an established diagnosis of CLL according to the criteria established by the international workshop on CLL^8^; (2) no indication for treatment at the time of diagnosis; and (3) Rai stage at diagnosis of 0, I, or II. Cases were defined as CLL patients with a TTFT of ≤24 months from sampling, whereas controls were defined as CLL patients with a TTFT exceeding 24 months from sampling, corresponding to previous definitions of “high-risk CLL.”^9^ For the mass spectrometry cohort, cases and controls were matched by sex and age at sampling (±2 years). For 10 patients, mass spectrometry was additionally performed on a repeated sample, obtained 6–12 months after the index sample.

### Sample preparation

Cryopreserved peripheral blood mononuclear cells (PBMCs) were thawed and 1 × 10^6^ cells were aliquoted for targeted fluorescence in-situ hybridization (FISH). For all other downstream purposes, B cells were purified from PBMCs using untouched magnetic activated cell sorting by negative selection (Human B Cell Isolation Kit II, Miltenyi Biotec, Bergisch Gladbach, Germany). As the B cell fraction of CLL patients is strongly dominated by leukemic cells, any contamination from healthy B cells in downstream analyses is negligible.

### Mass spectrometry

About 1 × 10^6^ sorted cells were washed 3 times with PBS and snap frozen in an ethanol and dry ice. Cell pellets were lysed in 1 mL 50 mM Tris/HCl pH 8.2, 0.5% sodium deoxycholate (SDC) using a Bioruptor ultasonicator (Diagenode, Liège, Belgium). Protein concentrations were measured using the bicinchoninic acid assay (BCA) assay (ThermoScientific). Proteins were reduced with 5 mM dithiothreitol and cysteine residues were alkylated with 10 mM iodoacetamide. Protein was extracted by acetone precipitation at −20°C overnight. Samples were centrifuged at 8000*g* for 10 minutes at 4°C. After acetone removal, the pellet was allowed to dry and dissolved in 1 mL 50 mM Tris/HCl pH 8.2, 0.5% SDC. Proteins were digested with LysC (1:200 enzyme:protein ratio) for 4 hours at 37°C. Next, trypsin was added (1:100 enzyme:protein ratio) and the digestion proceeded overnight at 30°C. Digests were acidified with 50 μL 10% formic acid and centrifuged at 8000*g* for 10 minutes at 4°C to remove the precipitated SDC. The supernatant was transferred to a new centrifuge tube. The digests were purified with C18 solid phase extraction (Sep-Pak, Waters Corporation, Milford, MA), lyophilized, and stored at −20°C. Each unlabeled sample was analyzed separately to check for the digestion efficiency before further tandem mass tag (TMT)-labeling.

Proteolytic peptides were then labeled with TMT 11-plex labeling reagents (ThermoScientific, Lot number UI281756) for 10 separate TMT liquid chromatography mass spectrometry (LC-MS) runs allowing for peptide quantitation. Peptides were mixed at the 11-plex level and analyzed by nanoflow LC-MS/MS. nLC-MS/MS was performed on EASY-nLC 1200 coupled to an Orbitrap Eclipse mass spectrometer (ThermoScientific) operating in positive mode and equipped with a nanospray source. Peptides were separated on a ReproSil C18 reversed phase column (Dr Maisch GmbH; column dimensions 15 cm × 50 µm, packed in-house) using a linear gradient from 0% to 80% B (A = 0.1% formic acid; B = 80% (v/v) acetonitrile, 0.1% formic acid) in 120 minutes and at a constant flow rate of 200 nL/min using a splitter. The column eluent was directly sprayed into the ESI source of the mass spectrometer. Field asymmetric waveform ion mobility spectrometry was used to separate peptides in the gas-phase (compensation voltages [CVs] used: 40, 50, 60, and 70). Each sample mix was analyzed 4 times at different CVs. Mass spectra were acquired in continuum mode; fragmentation of peptides was performed in data-dependent mode using the multinotch SPS MS3 reporter ion-based quantification method. Mass spectrometer settings, raw data processing, and quality control are detailed in the Supplemental Methods and Suppl. Table S1.

### Protein abundance analysis

Patient samples with ≥40% missingness or highly deviant values (>2 SD from the mean) were excluded from downstream analysis (Suppl. Figure S1). In addition, proteins with ≥50% missingness across all patients were excluded from downstream analysis. Protein abundance data were background corrected and scaled before analysis. For hierarchical clustering, missing values were imputed using the quantile regression imputation of left-censored data method using the imputeLCMD *R* package.^10^ To correct for batch effects, batch number was included as a covariate in all regression models and statistical tests. In addition, for data visualization, batch effects were reduced through the ComBat function in the *R* package sva.^11^ Data visualization was performed using the ggplot2 and pheatmap *R* packages.^12,13^

### Targeted FISH

FISH was carried out on fixated PBMCs to determine structural and numerical abnormalities involving chromosome 11q22 (LSI ATM, limit of detection [LOD] 8% for hemizygosity), chromosome 12 (CEP12, LOD 3% for trisomy), chromosome 13q14/13q34 (LSI D13S319, LOD 7% for hemizygosity), or chromosome 17p13 (LSI p53, LOD 10% for hemizygosity) (all Vysis, Abbott, Abbott Park, IL).

### IGHV, IGLV3-21, and TP53 mutational status profiling

Genomic DNA was isolated from up to 10^6^ sorted cells using the AllPrep DNA/RNA/miRNA Universal kit (Qiagen) and quantified using NanoDrop spectrophotometry (ThermoFisher). The leukemia-specific IGH rearrangement was amplified using IGHV-leader primers in a multiplex polymerase chain reaction (PCR), followed by family-specific amplification and Sanger sequencing using a BigDye Terminator v3.1 Cycle Sequencing Kit (ThermoFisher). For selected, difficult-to-solve cases, IGH rearrangements were amplified using the IGHV EuroClonality leader primer set,^14^ followed by NGS on a MiSeq using a v3 600 cycle kit (both Illumina, San Diego, CA) and analysis using the ARResT/Interrogate immunoprofiler.^15^ Nucleotide sequences were annotated using IMGT/V-QUEST.^16^ The IGHV mutational status was determined in accordance with the European Research Initiative on CLL recommendations.^17,18^ *TP53* mutations were detected by NGS as detailed previously,^19,20^ with a variant allele frequency cutoff of 10%. IGLV3-21^R110^ status was determined by Sanger sequencing of cDNA, as described previously.^21^

### Gene expression analysis

Total RNA was isolated from up to 10^6^ sorted cells according to the manufacturer’s protocol, using the AllPrep DNA/RNA/miRNA Universal kit (Qiagen) and quantified using NanoDrop spectrophotometry (ThermoFisher). cDNA was synthesized using SuperScript II reverse transcriptase (ThermoFisher). Real-time quantitative PCR (qPCR) of *THEMIS2* transcript was performed in triplicate using a TaqMan gene expression assay (ThermoFisher, ID: Hs00982814_g1), universal mastermix (Applied Biosystems, Waltham, MA) on a Quantstudio 3 (ThermoFisher). Replicates with a deviation of ≥0.25 cycle from the mean were excluded from the analysis. Cycle threshold values were normalized against expression of *ABL*.

### ELISA

For ELISA, up to 10^6^ sorted cells were lysed on ice in 100 μL RIPA buffer with 1% HALT phosphatase and protease inhibitor (both ThermoScientific, Waltham, MA). Protein concentration was quantified using a Pierce BCA protein assay kit (ThermoScientific). ELISA was performed according to the manufacturer’s protocol, using a Human Protein THEMIS2 ELISA kit (Abbexa Ltd, Cambridge, UK) with 500 ng of total protein content input in duplicate wells. Average optical density was measured at 450 nM and THEMIS2 concentration was interpolated using a standard curve generated from same-plate duplicate wells.

### Biomarker validation in external cohorts

Two publicly available, independently generated datasets were used for biomarker external validation. The first dataset, generated by Herbst et al, comprised bulk RNA sequencing and mass spectrometry data of 68 CLL patients.^22^ As not all patients in this cohort were treatment-free, treatment-free survival (TFS) was reported as end point. Kaplan-Meier survival curves were generated using the online tool available at www.dietrichlab.de/CLL_Proteomics/. The second dataset, published by Knisbacher et al, comprised bulk RNA sequencing, failure-free survival (FFS), and overall survival (OS) of 556 treatment-naive CLL patients. Clinical data and batch corrected transcript per million data was downloaded from www.CLLmap.org.

### Statistical analysis

The statistical analyses are described in the Supplemental Methods.

## RESULTS

### Mass spectrometry analysis of the proteome in untreated CLL

**Figure 1.**
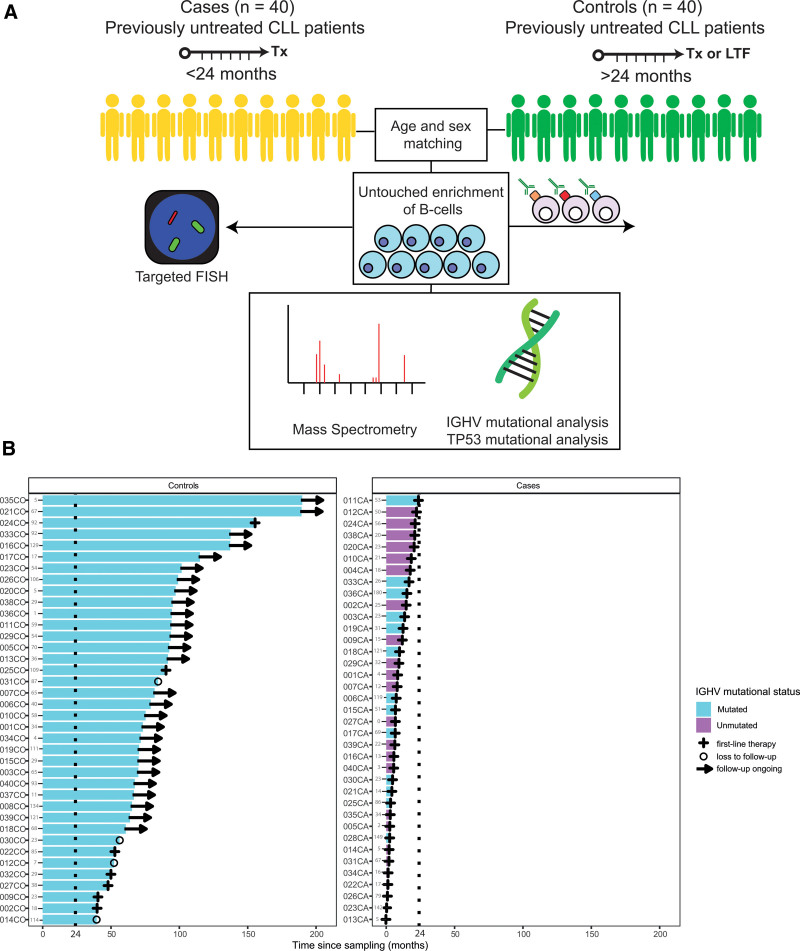
**Schematic representation of the study design.** (A) Schematic representation of the mass spectrometry screen. Forty cases (CLL patients with TTFT ≤24 mo) and 40 controls (CLL patients with TTFT >24 mo) were selected from the AUMC biobank and matched based on sex and age. B cells were purified from PBMCs using untouched magnetic sorting. Following purification, cells were aliquoted for downstream purposes. Targeted FISH was performed on unsorted PBMCs. (B) Swimmer plot illustrates TTFT of cases and controls in the mass spectrometry cohort. The dotted line indicates the cutoff at 24 mo, differentiating cases from controls. A cross indicates progression to first-line therapy, a circle indicates LTF, and an arrow indicates ongoing treatment-free survival. The interval (in months) between the moment of diagnosis and the moment of sampling is indicated in gray. AUMC = Amsterdam university medical centers; CLL = chronic lymphocytic leukemia, del; deletion; FISH = fluorescence in-situ hybridization; IGHV = immunoglobulin heavy-chain variable; LTF = loss to follow up; PBMCs = peripheral blood mononuclear cells; TTFT = time-to-first treatment.

To characterize global protein expression of untreated, early-stage CLL, we measured the intracellular protein abundances of magnetically purified B cells using mass spectrometry in a cohort of 40 CLL patients with TTFT ≤24 months after peripheral blood (PB) sampling (hereafter: cases) and 40 sex- and age-matched CLL patients with TTFT >24 months after PB sampling (hereafter: controls) (Figure 1A). Cases or controls with ≥40% missing data or highly deviant protein expression values (>2 SD from the mean), likely indicating technical artifacts, were excluded from the analysis (n = 5), resulting in a final cohort of 75 patients (Suppl. Figure S1; Suppl. Table S2). As expected, high-risk molecular features, such as unmutated IGHV, *TP53* anomalies, and IGLV3-21^R110^ were enriched in the cases. Median TTFT in the cases was 7.7 months versus not reached (NR) in the controls (Figure 1B).

In total, we quantified the expression of 3268 proteins in the cohort. Principal component analysis and unsupervised clustering by global protein expression did not show segregation by outcome or molecular features (Suppl. Figure S2A, B).

### Downstream protein correlates of CLL molecular features

**Figure 2.**
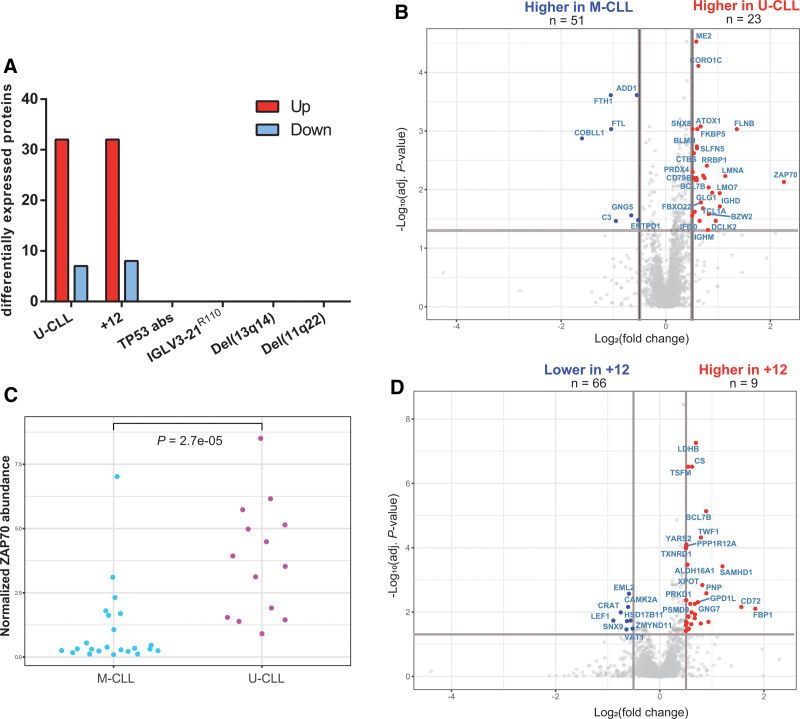
**Downstream proteomic effects of IGHV mutational status and trisomy 12.** (A) Bar chart represents the number of differentially expressed proteins (log_2_[fold change] ≥0.5 or ≤-0.5 and adjusted *P*-value <0.05) per molecular feature. (B) Volcano plot demonstrates for each protein the log_2_-transformed fold-change (x-axis) and adjusted *P*-value (y-axis), comparing unmutated CLL to mutated CLL. The gray lines illustrate the cutoffs for fold-change and statistical significance. Red and blue dots represent differentially expressed proteins. (C) Dot plot demonstrates the normalized relative intracellular abundance of ZAP70, stratified per IGHV mutational status. Fold-change values were normalized to the average abundance of M-CLL samples. Statistical significance was evaluated using a *t* test. (D) Volcano plot demonstrates for each protein the log_2_-transformed fold-change (x-axis) and adjusted *P*-value (y-axis), comparing CLL with trisomy 12 to all other samples. The gray lines illustrate the cutoffs for fold-change and statistical significance. Red and blue dots represent differentially expressed proteins. CLL = chronic lymphocytic leukemia; IGHV = immunoglobulin heavy-chain variable; M-CLL = CLL with mutated IGHV; U-CLL = CLL with unmutated IGHV.

To analyze the impact of well-known molecular characteristics of CLL on intracellular protein expression, we investigated differential protein expression (log_2_[fold change] ≥0.5 or ≤−0.5, adjusted *P*-value <0.05) associated with immunogenetic (IGHV mutational status and/or IGLV3-21^R110^ light chain use), cytogenetic, and genetic features (*TP53* mutations). In our analysis, IGHV mutational status (32 proteins overexpressed in U-CLL and 7 proteins overexpressed in M-CLL) and trisomy 12 (32 proteins overexpressed in the presence of trisomy 12 and 8 proteins overexpressed in the absence of trisomy 12) were most impactful on the proteome (Figure 2A; Suppl. Tables S3 and S4). *TP53* aberrancies (ie, del(17p13) and/or *TP53* mutations), IGLV3-21^R110^, del(13q14), and del(11q22) were not associated with differential protein expression (Figure 2A).

Comparing U-CLL to M-CLL, the highest fold-change was observed for ZAP70, with a 4.7-fold higher average abundance in U-CLL, compared with M-CLL (Figure 2B, C). High expression of ZAP70 is a well-known feature of U-CLL, thus serving as a validation of our dataset. In addition, abundance of the IG constant region mu and delta (IGHM and IGHD) and CD79B, part of the B cell receptor (BCR) complex, was higher in U-CLL, in concordance with the higher surface IgM expression and BCR responsiveness observed in these patients.^23,24^ In contrast to previous research,^25^ MARCKS was not differentially abundant between U-CLL and M-CLL patients in our cohort (Suppl. Figure S3A). Notably, 13 of the 32 (41%) proteins overexpressed in CLL with trisomy 12 were encoded by genes on chromosome 12, indicative of gene dosage effects (Figure 2D).

### Cross-validation with publicly available mass spectrometry screens in CLL cohort

**Figure 3.**
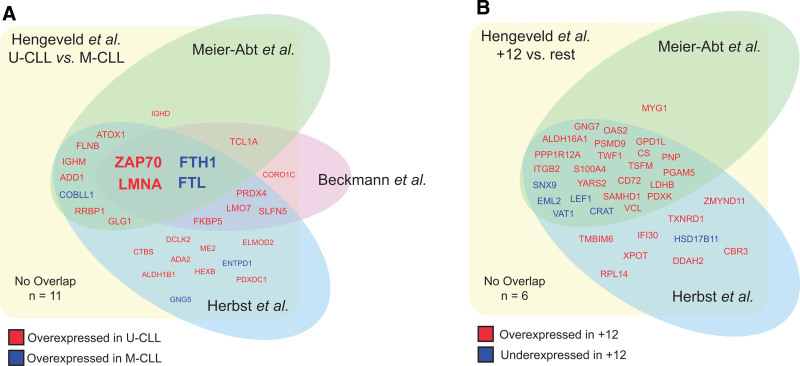
**Reproducibility of mass spectrometry screens in CLL.** (A) Venn diagram demonstrates the overlap in differentially expressed proteins between U-CLL and M-CLL, comparing the results of our mass spectrometry screens to 3 recently performed screens from the literature. Proteins in red were overexpressed in U-CLL, whereas proteins in blue where overexpressed in M-CLL. (B) Venn diagram demonstrates the overlap in differentially expressed proteins between CLL with trisomy 12 and all other samples, comparing the results of our mass spectrometry screens to 3 recently performed screens from the literature. Proteins in red were overexpressed in CLL with trisomy 12, whereas proteins in blue where underexpressed in CLL with trisomy 12. whereas proteins in blue where underexpressed in CLL with trisomy 12. CLL = chronic lymphocytic leukemia; M-CLL = CLL with mutated IGHV; U-CLL = CLL with unmutated IGHV.

Three other mass spectrometry screens of the CLL proteome have recently been published.^22,25,26^ To investigate the validity and reproducibility of mass spectrometry screens in CLL, we compared our findings with the differentially expressed proteins (DEPs) reported in these screens. Indeed, in spite of the differences in study design and methodology, a large number of DEPs found in our screen were concordantly reported in one or multiple other screens. Specifically, 28 of 39 (72%) DEPs associated with IGHV mutational status (Figure 3A) and 34 of 40 (85%) DEPs associated with trisomy 12 (Figure 3B) were found both in our screen, and in 1 or more screens from the literature, in all cases sharing directionality. Regarding IGHV mutational status, 4 proteins were consistently identified as differentially expressed in all 4 screens: ZAP70 and Lamin type A/C, overexpressed in U-CLL, and FTH1 and FTL, both overexpressed in M-CLL.

### Biomarker identification in untreated CLL

**Figure 4.**
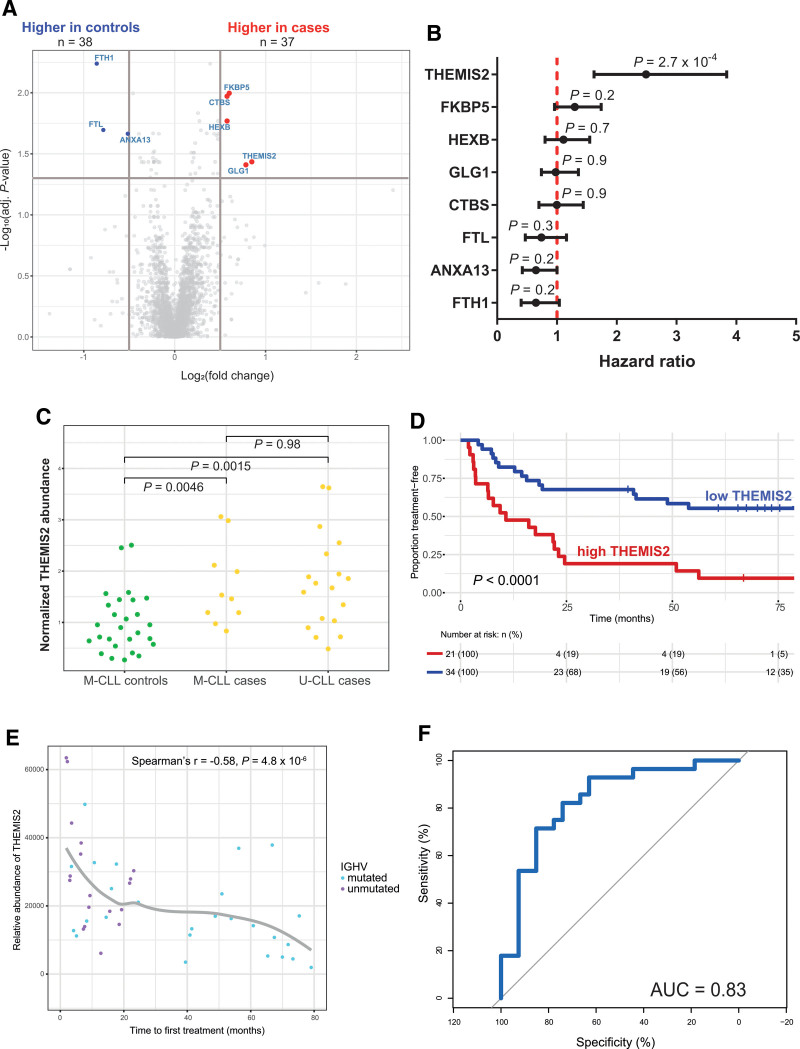
**Identification of THEMIS2 as putative biomarker for TTFT.** (A) Volcano plot demonstrates for each protein the log_2_-transformed fold-change (x-axis) and adjusted *P*-value (y-axis), comparing cases to controls. The gray lines illustrate the cutoffs for fold-change and statistical significance. Red and blue dots represent differentially expressed proteins. (B) Dot-and-whisker plot represents the hazard ratio per protein in a Cox proportional hazards model, estimating TTFT with IGHV and TP53 mutation status as covariables. Dots represent the point estimate, whereas the whiskers extend to the upper and lower limit of the 95% confidence interval. Statistical significance was evaluated using a Wald test. (C) Dot plot demonstrates the normalized relative intracellular abundance of THEMIS2, stratified by case/control identity and IGHV mutational status. Fold-change values were normalized to the average abundance of M-CLL controls. Statistical significance was evaluated using a *t* test. (D) Kaplan-Meier survival plot estimates the proportion of treatment-free patients over time, stratified by above or below average intracellular abundance of THEMIS2 protein. Asterisks indicate right-censoring. Statistical significance was evaluated using a log-rank test. (E) Correlation plot, relating TTFT to the relative abundance of THEMIS2 obtained by mass spectrometry. Each dot represents a patient. A regression line (gray) was plotted using LOESS. (F) Receiver-operating characteristic analysis demonstrates the specificity and sensitivity for discrimination between cases and controls for each threshold value of THEMIS2 relative intracellular abundance in blue. The AUC indicates the discriminator capacity. An AUC of 0.50 (gray line) indicates discriminatory capacity equal to random chance. AUC = area under the curve; CLL = chronic lymphocytic leukemia; IGHV = immunoglobulin heavy-chain variable; LOESS = locally estimated scatterplot smoothing; M-CLL = CLL with mutated IGHV; TTFT = time-to-first treatment; U-CLL = CLL with unmutated IGHV.

When comparing the DEPs between cases and controls, 5 proteins were significantly upregulated in the cases, whereas 3 proteins were significantly downregulated (Figure 4A). Inevitably, many of these DEPs were correlated to IGHV mutational status, as U-CLL was exclusively present among the cases. To identify which of these proteins may aid prognostication beyond IGHV and *TP53* mutational status, we performed Cox PH modeling, estimating the hazard of progression to therapy as a function of protein expression, while correcting for IGHV mutational status and *TP53* aberrancies. Of all 8 proteins, only elevated protein expression of THEMIS2 was independently associated with TTFT (hazard ratio, 2.98 [95% confidence interval (CI), 1.87-4.73]; *P* < 0.001) (Figure 4B). Indeed, average THEMIS2 protein levels were elevated in both U-CLL and M-CLL cases, compared with controls, which were all M-CLL (Figure 4C), and was unrelated to the presence of IGLV3-21^R110^ (Suppl. Figure S3B). High intracellular protein levels of THEMIS2 were associated with shorter TTFT (relative THEMIS2 expression >mean, median TTFT 10.7 months [95% CI, 6.4-24.6] versus <mean, median TTFT NR [95% CI, 41.4-NR]; *P* < 0.001) and FFS (relative THEMIS2 expression >mean, median FFS 9.3 months [95% CI, 6.6-NR] versus <mean, median FFS 44.2 [95% CI, 14.43-NR]; *P* = 0.0019) (Figure 4D; Suppl. Figure S3C). An increase in THEMIS2 protein abundance was inversely correlated to TTFT (Spearman’s *r*= −0.58; *P* < 0.001) (Figure 4E). Receiver-operating characteristic analysis demonstrated good discriminatory capacity of relative THEMIS2 protein levels, distinguishing cases from controls with an area under the curve of 0.83 (Figure 4F). For 10 patients, a follow-up sample was available, obtained 6–12 months after the index sample. In this interval, none of these patients received treatment. THEMIS2 protein levels, measured by mass spectrometry, were stable over time, showing strong correlation between measurements (r = 0.73, [95% CI, 0.18-0.93]; *P* = 0.02) (Suppl. Figure S3D).

### Additive value of THEMIS2 to the IPS-E index

The International Prognostic Score for Early-stage CLL (IPS-E) index is a prediction model that estimates TTFT in early-stage CLL based on the IGHV mutational status, an absolute lymphocyte count (ALC) >15 × 10^9^/L and lymph node palpability.^27^ Addition of THEMIS2 as predictor variable in a Cox PH model including IGHV mutational status, ALC and Rai stage at diagnosis, as surrogate for lymph node involvement, significantly improved model goodness-of-fit (likelihood ratio test; *P* < 0.001) (Suppl. Table S5).

### Measurement of THEMIS2 expression by qPCR and ELISA

**Figure 5.**
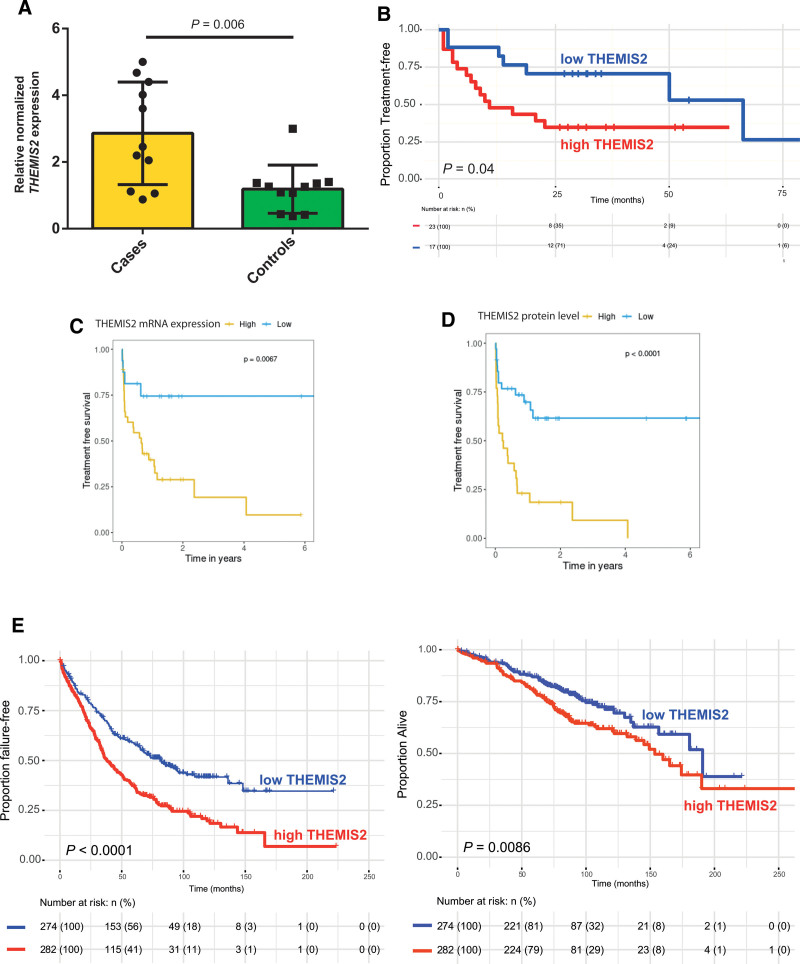
**High intracellular THEMIS2 as putative biomarker for inferior TTFT.** (A) Dot plot and bar chart represent the relative normalized gene expression of *THEMIS2* in cases and controls. Cycle threshold values were corrected for expression of *ABL* and normalized to the average gene expression levels in control patients. Statistical significance was evaluated using a *t* test. (B) Kaplan-Meier survival plot estimates the proportion of treatment-free patients over time, stratified by above or below average intracellular levels of THEMIS2 protein, quantified by ELISA. Asterisks indicate right-censoring. Statistical significance was evaluated using a log-rank test. (C and D) Kaplan-Meier survival plots estimate the proportion of treatment-free patients over time, stratified by above or below average *THEMIS2* gene expression (C) or THEMIS2 protein expression (D). Statistical significance was evaluated using a log-rank test. These figures were generated using an external validation cohort, through the online application kindly provided by Herbst et al, available at https://www.dietrichlab.de/CLL_Proteomics/. (E and F) Kaplan-Meier survival plots estimate the proportion of failure-free (C) or alive (D) patients over time, stratified by above or below average *THEMIS2* gene expression. Statistical significance was evaluated using a log-rank test. These figures were generated using an external validation cohort published by Knisbacher et al. These figures were generated using an external validation cohort published by Knisbacher et al. CLL = chronic lymphocytic leukemia; ELISA = enzyme-linked immunosorbent assay; TTFT = time-to-first treatment.

THEMIS2, which functions downstream of the BCR, increases BCR sensitivity to low-avidity antigens and increases BCR activation in the absence of antigen.^28^ To confirm the relationship between THEMIS2 and TTFT, we measured *THEMIS2* gene expression by qPCR in cases and controls. Average normalized expression of *THEMIS2* mRNA was 2.9-fold higher in cases, compared with controls (*P* = 0.005) (Figure 5A). Furthermore, direct quantification of THEMIS2 by ELISA in a cohort of 40 patients (21 patients from the mass spectrometry screen and 19 new patients; Suppl. Table S6) demonstrated that higher intracellular concentrations of THEMIS2 were associated with shorter TTFT (THEMIS2 concentration >mean [>1166 pg/mL], median TTFT 11 months [95% CI, 7-NR], versus <mean [<1166 pg/mL], 66 months [95% CI, 50-NR]; *P* = 0.04) (Figure 5B).

### External validation of THEMIS2 as putative biomarker for short TTFT

We evaluated the prognostic utility of THEMIS2 in 2 publicly available, independently generated datasets. The first, generated by Herbst et al, encompasses bulk RNA sequencing and mass spectrometry data of 68 CLL patients.^22^ As not all patients in this cohort were treatment-naïve, TFS rather than TTFT was reported. In this cohort, elevated expression of THEMIS2 was associated with inferior TFS, both on the mRNA level (high expression, median TFS ≈10 months, versus low expression, median TFS NR; *P* = 0.007) (Figure 5C) and on the protein level (high abundance, median TFS ≈4 months, versus low abundance, median TFS NR; *P* < 0.0001) (Figure 5D).^22^ In the second dataset, assembled by Knisbacher et al, bulk RNA sequencing and clinical data were available for 556 treatment-naïve CLL patients.^29^ Baseline characteristics of these patients are listed in Suppl. Table S7. In these patients, high gene expression of *THEMIS2* was associated with significantly shorter FFS and OS (median FFS, *THEMIS2* expression >mean 38.2 months [95% CI, 33.6-49] versus <mean, 81.8 months [95% CI, 65.1-109]; *P*<0.001 and median OS, *THEMIS2* expression >mean 154 months [95% CI, 139-NR] versus 191 months [95% CI, 157-NR]; *P* = 0.009) (Figure 5E, F).^29^ In a Cox PH model, including IGHV mutational status and presence or absence of *TP53* aberrations and IGLV3-21^R110^ as covariables, *THEMIS2* gene expression independently predicted FFS, but not OS (Suppl. Tables S8 and S9).

## DISCUSSION

In this study, we have characterized the global intracellular proteome of 75 early-stage, treatment-naïve CLL patients. We describe the downstream effects on protein expression of recurrent molecular features of CLL and use the unique case-control design of our early-stage cohort to identify THEMIS2 as novel, independent biomarker associated with TTFT at the time of sampling.

THEMIS2, alternatively known as ICB-1, is a newly described member of the Themis family of proteins. Although THEMIS1 plays a nonredundant role in positive selection of CD8^+^CD4^+^ T cells in the thymus, THEMIS2 is mainly expressed in monocytes, follicular B cells, and B1 B cells.^28,30^ The latter observation is noteworthy, as human B1-like B cells have been proposed to be the cell of origin in CLL.^31^ Although THEMIS2 is not essential for B cell biology, it increases BCR sensitivity to low-avidity, but not high-avidity antigens by constitutive binding to Grb2, Lyn, and PLCγ2.^28^ Crucially, THEMIS2 may increase constitutive BCR activation in the absence of antigen.^28^ Whether the relationship between THEMIS2 and survival is purely associative, or whether THEMIS2 may play a role in the pathophysiology of CLL, remains to be determined.

The association between THEMIS2 and outcome was present at both mRNA and protein level, as demonstrated by qPCR, mass spectrometry, and ELISA. External validation was performed by analysis of 2 independently generated datasets, published by Herbst et al and Knisbacher et al, which demonstrated an inverse association between *THEMIS2* gene and protein expression and TFS, FFS, and OS.^22,29^ Of note, the association between THEMIS2 and TTFT or FFS was independent of IGHV mutational status and *TP53* aberrancies. Moreover, incorporation of THEMIS2 protein levels in a model containing all components of the IPS-E index,^27^ a validated prognostic model in the context of early-stage CLL, improved model performance. As such, high intracellular THEMIS2 concentration, as measured by clinically applicable techniques such as ELISA, could be putatively positioned as biomarker of poor prognosis in early-stage CLL, complementary to IGHV and *TP53* mutational profiling. Defining the optimal measurement modality and threshold value for THEMIS2-based prognostication requires additional work in a larger cohort of patients.

Recently, several mass spectrometry screens have been published in the context of CLL. Beckmann et al compared the proteome and phosphoproteome in a small series of 3 U-CLL and 3 M-CLL patients, using isobaric tags for relative and absolute quantification-based mass spectrometry.^25^ Meier-Abt et al used data-independent acquisition-mass spectrometry to characterize the proteome of 117 CLL patients.^26^ Lastly, Herbst et al, using in-depth high-resolution isoelectric-mass spectrometry, characterized protein expression in a cohort of 68 CLL patients.^22^ Comparing the result of these contemporaneous, independently performed mass spectrometry screens allows for an assessment of the validity and reproducibility of proteome profiling in CLL.

There was high concordance between all 4 reported screens. Similarly to Meier-Abt et al and Herbst et al, in our screen, the molecular features with the highest impact on the proteome were IGHV mutational status and trisomy 12.^22,27^ The proteomic effects of other features, specifically IGLV3-21^R110^ light chain use, del(13q14), del(11q22), and *TP53* aberrancies, were negligible. Importantly, 72% of DEPs associated with IGHV mutational status and 85% of DEPs associated with trisomy 12 identified in our analysis were reported in at least 1 of the 3 screens from the literature, underscoring the robust nature of these mass spectrometry screens from a Bayesian perspective.

Differential expression of 4 proteins was consistently associated with IGHV mutational status across all 4 screens: ZAP70 and Lamin type A/C, with high expression in U-CLL, and the heavy and light chain of ferritin, with high expression in M-CLL. Although the association between ZAP70 expression and IGHV mutational status has been widely established, to our knowledge, the role of these other proteins has not been studied in the context of CLL. Lamin A/C belongs to the lamin family of proteins, components of the nuclear envelope. Lamin B1, a similar perinuclear protein, has been implicated in the regulation of SHM by binding to the immunoglobulin domains: *LMNB1* expression is decreased in germinal centers, induction of SHM lowers Lamin B1 expression and suppression of Lamin B1 grossly increases SHM rates.^32^ Of note, in contrast to Lamin type A/C, *LMNB1* expression was upregulated in M-CLL, and low *LMNB1* expression was associated with inferior survival.^32^ Whether LMNA is similarly involved in the molecular machinery of SHM warrants further investigation. The heavy and light chains of ferritin, FTH1 and FTL, form polymers of 24 subunits to comprise ferritin, which is responsible for the intracellular sequestration of iron. Free intracellular iron is cytotoxic, as it catalyzes the formation of free radicals in the Fenton reaction. As such, the high expression of ferritin may, speculatively, render M-CLL cells differentially susceptible to ferroptosis. Of note, a recent study correlated the expression of eight ferroptosis-related genes to the clinical outcome of CLL patients, and to the predicted in vitro susceptibility of CLL cells to fludarabine, cyclophosphamide, and ibrutinib, further indicating that ferroptosis may play an important role in the context of CLL.^33^

Our study has some limitations. First, we confined the molecular characterization of our cohort to those features commonly determined in clinical practice. As such, we were unable to study the proteomic effects of other recurrent mutations, such as lesions affecting *NOTCH1* and *SF3B1*, although in other screens, their effects on global protein expression were found to be minimal.^22,26^ Second, as biobank participation terminated upon treatment initiation, the effects of protein expression on treatment efficacy and OS could not be evaluated in this CLL biobank cohort. In addition, several patients in the cases group rapidly progress to treatment. In these patients, our use of THEMIS2 expression within the framework of the IPS-E index should be interpreted with caution, as this scoring system is developed for use in early-stage disease (Binet A). Finally, our cohort was powered and matched to evaluate differences using a TTFT threshold of 24 months. Consequently, proteins identified as DEPs at this threshold might not have the same discriminatory capacity at shorter (ie, 6–12 months) or longer (ie, 36 or 48 months) TTFT intervals. That said, our survival analyses demonstrate that the discriminatory capacity of THEMIS2 persists across the entire length of follow-up time in our cohort.

In summary, we provide a comprehensive characterization of the global proteome of early-stage CLL. We find that high expression of THEMIS2 is a biomarker that, independently of IGHV mutational status or TP53 aberrations, predicts short TTFT at the time of sampling.

## ACKNOWLEDGMENTS

The authors thank all patients, their families, and investigators involved in the biobank for B cell malignancies. In addition, we thank Abt-Meier et al, Herbst et al, and Knischbacher et al for making their datasets publicly available as interactive web modules. This project was partly funded by the 2021 Albert Schweitzer Hospital Stipend for Research.

## AUTHOR CONTRIBUTIONS

PJH, M-DL, and AWL contributed to study design. PJH performed the experimental procedures. PMK contributed to data analysis and interpretation. JAAD and WD performed the mass spectrometry and contributed to data analysis. JMND and APK were involved in the coordination of the biobank for B cell malignancies. MR performed the *TP53* sequencing. HJB performed the IG light chain sequencing. KJG performed the targeted FISH. JLJCA, LvdS, PJMV, and PEW contributed to data interpretation. PJH, PMK, M-DL, and AWL wrote the article. All authors critically read and approved the final version of the article.

## DATA AVAILABILITY

The mass spectrometry proteomics data have been deposited to the ProteomeXchange Consortium via the PRIDE partner repository, with the dataset identifier PXD036918. The scripts used for the bioinformatic analyses have been uploaded to https://github.com/paul10011993/CLLproteome.

## DISCLOSURES

JMND has received research funding from Roche/Genentech. APK has received personal fees from AbbVie, LAVA, Genmab, Janssen, AstraZeneca, Roche/Genentech, and Bristol Myers Squibb; and research funding from AbbVie, Janssen, AstraZeneca, Roche/Genentech, and Bristol Myers Squibb. M-DL has received personal fees from AbbVie, Janssen, and Roche; and research funding from AbbVie, Janssen, AstraZeneca, and Roche/Genentech. AWL has received research funding via an unrestricted grant from Roche-Genentech and speaker-fees from Janssen. All the other authors have no conflicts of interest to disclose.

## SOURCES OF FUNDING

The authors declare no (external) sources of funding.

## Supplementary Material


