# Supplementary material for: High-throughput Proteomics Identifies THEMIS2 as Independent Biomarker of Treatment-free Survival in Untreated CLL
Source: Hemasphere. 2023 Sep 15;7(10):e951. doi: 10.1097/HS9.0000000000000951 (PMC10508458; doi:10.1097/HS9.0000000000000951)
Supplement: Supplementary file 1 [file hs9-7-e951-s001.docx]

**Supplementary Methods**

*Mass spectrometry settings*

The global method settings were as follows. Master scan: desired minimum points across the peak = 9; msn level = 1; use wide quad isolation = true; detector type = orbitrap; orbitrap resolution = 120K; mass range = normal; scan range (m/z) = 400-1600; maximum injection time (ms) = 50; AGC target = 400000; normalized AGC target = 100%; microscans = 1; maximum injection time type = auto; RF lens (%) = 30; use ETD internal calibration = false; FAIMS voltages on = true; datatype = profile; FAIMS CV = -40 or -50 or -60 or -70; polarity = positive; source fragmentation = false; scan description = enhanced resolution mode = off; filter precursor selection range; mass range = 400-1600; filter charge state; include charge state(s) = 2-6; include undetermined charge states = false; filter dynamic exclusion; exclude after n times = 1; exclusion duration (s) = 45; mass tolerance = ppm; mass tolerance low = 10; mass tolerance high = 10; use common settings = false; exclude isotopes = true; perform dependent scan on single charge state per precursor only = true; filter intensity threshold; maximum intensity = 1e+20; minimum intensity = 5000; relative intensity threshold = 0; intensity filter type = intensity threshold; filter precursor fit: fit error (%) = 50; fit window = 1.2.

For the data dependent properties MSn level was set to 2 and the isolation mode as quadrupole. further settings: enable intelligent product acquisition for MS2 isolation = false; isolation window = 1.2; isolation offset = off; reported mass = original mass; multi-notch isolation = false; scan range mode = auto; scan priority= 1; collision energy mode = fixed; activation type = CID; collision energy (%) = 35; activation time (ms) = 10; activation q = 0.25; multistage activation = false; detector type = iontrap; ion trap scan rate = turbo; maximum injection time (ms) = 35; AGC target = 10000; inject ions for all available parallelizable time = false; normalized AGC target = 100%; microscans = 1; maximum injection time type = custom; use ETD internal calibration = false; datatype = centroid; polarity = positive; source fragmentation = false; scan description = ; time mode = unscheduled; enhanced resolution mode = off; filter precursor selection range MSn; mass range = 400-1600; range relative to parent mass (%) = 0-1000; mass tolerance unit = mz; filter isobaric tag exclusion; exclusion mass tolerance is unknown; low 0; high 0; reagent tag type = TMT; filter precursor ion exclusion; mass tolerance is ppm; low 25; high 25; filter real time search filter; fragment ion tolerance (Da) = 0; end triggering after max number of peptides found = false; max number of peptides per protein = 4; enzyme = trypsin; SPS mode = true; precursor neutral loss = 0,0; enzyme specificity = full; decoy search mode = false; max missed cleavages = 1; max variable mods = 2; max search time (ms) = 100; use as trigger only = false; custom fragment ions = ; use custom fragment ions = false. For the data dependent properties, the mode ‘scans per outcome’ was chosen. Further settings: desired minimum points across the peak = 9; MSn level = 3; isolation mode = quadrupole; isolation offset = off; isolation window = 0.7; enable intelligent product acquisition for MS2 isolation = false; reported mass = original mass; multi-notch isolation = true; MS2 isolation window (m/z) = 2; number of notches = 10; scan range mode = define m/z range; scan priority= 1; activation type = HCD; collision energy mode = fixed; collision energy (%) = 65; detector type = orbitrap; orbitrap resolution = 50K; scan range (m/z) = 100-500; maximum injection time (ms) = 200; AGC target = 100000; inject ions for all available parallelizable time = false; normalized AGC target = 200%; microscans = 1; maximum injection time type = custom; use ETD internal calibration = false; datatype = centroid; polarity = positive; source fragmentation = false; scan description = ; time mode = unscheduled; enhanced resolution mode = off.

*Raw data processing and quality control*

Mass spectrometry data were analyzed with Proteome Discoverer 2.5 (ThermoScientific). The Mascot search algorithm (version 2.3.2, MatrixScience) was used for searching against the Uniprot database (taxonomy: Homo sapiens, version 06-2021). The peptide tolerance was typically set to 10ppm and the fragment ion tolerance was set to 0.6Da. The integration tolerance for reporter ion quantitation was set to 20ppm. A maximum number of 2 missed cleavages by trypsin were allowed and carbamidomethylation on cysteine was set as fixed modification, while oxidation on methionine and TOMT on lysine side chains and peptide N-termini were set as variable modifications. Relative abdundances of proteins were determined using the median of TMT reporter ion intensity ratios from all PSMs matching to the protein or phosphorylation site. PSMs having a precursor ion purity <50% were excluded from further analysis. Isotope corrections for TMT labels were applied according to the manufacturer’s recommendation. A pooled sample composed of equal fractions of all samples in the cohort was used as an internal reference standard (IRS; TMT channel 126 in all runs) for inter-run normalization of peptide abundances. Raw reporter ion abundances were extracted from PD2.5 and normalization of peptide abundances between the separate TMT-plexed LC-MS runs and further analysis was performed in *R*. An overview of TMT labeling is provided in **Supplementary Table 1**.

*Statistical analysis*

To test for associations between molecular features and intracellular protein abundances, we used a two-way ANOVA, including protein abundance as the quantitative variable with the molecular feature and batch number as independent categorical variables. All *P*-values were adjusted for multiple testing, using the Benjamini-Hochberg method. Differences in mean expression of individual proteins and difference in gene expression levels were compared using unpaired *t-*tests. TTFT and TFS were defined as the interval between the date of sampling and the initiation of first-line or next line of therapy, respectively. Failure-free survival (FFS) was defined as the interval between date of sampling and the initiation of the next line of therapy or death. Overall survival (OS) was defined as the interval between date of sampling and death. Univariable survival analysis was performed using Kaplan-Meier estimation, evaluating statistical significance using a log-rank test. Multivariable survival analysis was performed using Cox proportional hazards modelling. Hazard ratios were evaluated for statistical significance using a Wald test with correction for multiple testing. All survival analysis was performed in *R*, using the survival package(1). Model goodness-of-fit was compared using a likelihood ratio test.

**Supplementary Tables**

**Supplementary Table 1.** TMT-labeling overview

|  |  |  | TMT11plex | label |
| --- | --- | --- | --- | --- |
|  |  | sample # |  |  |
| mix 1 | pool 1 |  | pool | 126 |
|  | 2 | 1 | 001CA | 127N |
|  | 3 | 2 | 001CO | 127C |
|  | 4 | 3 | 002CA | 128N |
|  | 5 | 4 | 002CO | 128C |
|  | 6 | 5 | 003CA | 129N |
|  | 7 | 6 | 003CA2 | 129C |
|  | 8 | 7 | 003CO | 130N |
|  | 9 | 8 | 003CO2 | 130C |
|  | 10 | 55 | 022CA | 131 |
|  | 11 | 56 | 022CO | 131C |
| mix 2 | pool 1 |  | pool | 126 |
|  | 2 | 15 | 006CA | 127N |
|  | 3 | 16 | 006CO | 127C |
|  | 4 | 17 | 007CA | 128N |
|  | 5 | 18 | 007CO | 128C |
|  | 6 | 19 | 008CA | 129N |
|  | 7 | 20 | 008CO | 129C |
|  | 8 | 21 | 009CA | 130N |
|  | 9 | 22 | 009CA2 | 130C |
|  | 10 | 23 | 009CO | 131 |
|  | 11 | 24 | 009CO2 | 131C |
| mix 3 | pool 1 |  | pool | 126 |
|  | 2 | 9 | 004CA | 127N |
|  | 3 | 10 | 004CA2 | 127C |
|  | 4 | 11 | 004CO | 128N |
|  | 5 | 12 | 004CO2 | 128C |
|  | 6 | 13 | 005CA | 129N |
|  | 7 | 14 | 005CO | 129C |
|  | 8 | 25 | 010CA | 130N |
|  | 9 | 26 | 010CO | 130C |
|  | 10 | 27 | 011CA | 131 |
|  | 11 | 28 | 011CO | 131C |
| mix 4 | pool 1 |  | pool | 126 |
|  | 2 | 29 | 012CA | 127N |
|  | 3 | 30 | 012CA2 | 127C |
|  | 4 | 31 | 012CO | 128N |
|  | 5 | 32 | 012CO2 | 128C |
|  | 6 | 33 | 013CA | 129N |
|  | 7 | 34 | 013CO | 129C |
|  | 8 | 35 | 014CA | 130N |
|  | 9 | 36 | 014CO | 130C |
|  | 10 | 37 | 015CA | 131 |
|  | 11 | 38 | 015CO | 131C |
| mix 5 | pool 1 |  | pool | 126 |
|  | 2 | 45 | 018CA | 127N |
|  | 3 | 46 | 018CO | 127C |
|  | 4 | 47 | 019CA | 128N |
|  | 5 | 48 | 019CA2 | 128C |
|  | 6 | 49 | 019CO | 129N |
|  | 7 | 50 | 019CO2 | 129C |
|  | 8 | 51 | 020CA | 130N |
|  | 9 | 52 | 020CO | 130C |
|  | 10 | 53 | 021CA | 131 |
|  | 11 | 54 | 021CO | 131C |
| mix 6 | pool 1 |  | pool | 126 |
|  | 2 | 59 | 024CA | 127N |
|  | 3 | 60 | 024CA2 | 127C |
|  | 4 | 61 | 024CO | 128N |
|  | 5 | 62 | 024CO2 | 128C |
|  | 6 | 63 | 025CA | 129N |
|  | 7 | 64 | 025CO | 129C |
|  | 8 | 65 | 026CA | 130N |
|  | 9 | 66 | 026CO | 130C |
|  | 10 | 67 | 027CA | 131 |
|  | 11 | 68 | 027CO | 131C |
| mix 7 | pool 1 |  | pool | 126 |
|  | 2 | 75 | 030CA | 127N |
|  | 3 | 76 | 030CO | 127C |
|  | 4 | 77 | 031CA | 128N |
|  | 5 | 78 | 031CO | 128C |
|  | 6 | 79 | 032CA | 129N |
|  | 7 | 80 | 032CO | 129C |
|  | 8 | 81 | 033CA | 130N |
|  | 9 | 82 | 033CA2 | 130C |
|  | 10 | 83 | 033CO | 131 |
|  | 11 | 84 | 033CO2 | 131C |
| mix 8 | pool 1 |  | pool | 126 |
|  | 2 | 69 | 028CA | 127N |
|  | 3 | 70 | 028CO | 127C |
|  | 4 | 89 | 036CA | 128N |
|  | 5 | 90 | 036CO | 128C |
|  | 6 | 91 | 037CA | 129N |
|  | 7 | 92 | 037CO | 129C |
|  | 8 | 93 | 038CA | 130N |
|  | 9 | 94 | 038CO | 130C |
|  | 10 | 95 | 039CA | 131 |
|  | 11 | 96 | 039CO | 131C |
| mix 9 | pool 1 |  | pool | 126 |
|  | 2 | 39 | 016CA | 127N |
|  | 3 | 40 | 016CO | 127C |
|  | 4 | 41 | 017CA | 128N |
|  | 5 | 42 | 017CA2 | 128C |
|  | 6 | 43 | 017CO | 129N |
|  | 7 | 44 | 017CO2 | 129C |
|  | 8 | 97 | 040CA | 130N |
|  | 9 | 98 | 040CA2 | 130C |
|  | 10 | 99 | 040CO | 131 |
|  | 11 | 100 | 040CO2 | 131C |
| mix 10 | pool 1 |  | pool | 126 |
|  | 2 | 57 | 023CA | 127N |
|  | 3 | 58 | 023CO | 127C |
|  | 4 | 71 | 029CA | 128N |
|  | 5 | 72 | 029CA2 | 128C |
|  | 6 | 73 | 029CO | 129N |
|  | 7 | 74 | 029CO2 | 129C |
|  | 8 | 85 | 034CA | 130N |
|  | 9 | 86 | 034CO | 130C |
|  | 10 | 87 | 035CA | 131 |
|  | 11 | 88 | 035CO | 131C |

**Supplementary Table 2.** Baseline characteristics of the CLL cohort used for mass spectrometry analysis

|  | **Cases (n = 37)** | **Controls (n = 38)** |
| --- | --- | --- |
| Sex (n, %)  *Male*  *Female* | 22 (63%)  15 (37%) | 24 (60%)  14 (40%) |
| Age (years)  *Median (IQR)* | 64 (59-68) | 63 (59-68) |
| RAI stage at diagnosis (n, %)  *0*  *I*  *II* | 20 (54%)  11 (30%)  6 (16%) | 29 (76%)  6 (15%)  3 (8%) |
| IGHV mutational status (n, %)  *Unmutated*  *Mutated* | 23 (62%)  14 (38%) | 0 (0%)  38 (100%) |
| Cytogenetic aberrations (n, %)  *del(13q14)*  *del(11q22)*  *del(17p13)*  *trisomy 12* | 24 (65%)  4 (11%)  6 (16%)  7 (19%) | 24 (63%)  1 (3%)  1 (3%)  2 (5%) |
| TP53 mutations (n, %)  *Present*  *Absent*  *Missing* | 6 (16%)  31 (84%)  0 (0%) | 3 (8%)  34 (89%)  1 (3%) |
| IGLV3-21^R110^  *Present* | 6 (16%) | 0 (0%) |

**Supplementary Table 5.** Cox proportional hazard model including IGHV mutational status, absolute lymphocyte count, lymph node involvement and THEMIS2 protein expression. This model is based on the IPS-E index(2).

| **Time to first treatment** | **HR [±SE]** | **P-value** |
| --- | --- | --- |
| THEMIS2 gene expression  *per SD increase* | 2.36 [±0.22] | **<0.0001** |
| IGHV mutational status  *M-CLL*  *U-CLL* | -  5.01 [±0.44] | -  **0.0003** |
| Absolute lymphocyte count  *≤15*10^9^/L*  *>15*10^9^/L* | -  2.02 [±0.79] | -  0.4 |
| Rai stage at diagnosis*  *0*  *I or II* | -  1.7 [±0.37] | -  0.2 |

*Rai stage is used as a proxy for lymph node palpability

**Supplementary Table 6.** Baseline characteristics of the CLL cohort used for ELISA

|  | **Cases (n = 20)** | **Controls (n = 20)** |
| --- | --- | --- |
| Included in screen (n, %)  *Yes*  *No* | 15 (75%)  5 (25%) | 6 (30%)  14 (70%) |
| RAI stage at diagnosis (n, %)  *0*  *I*  *II*  *missing* | 9 (45%)  4 (20%)  5 (25%)  2 (10%) | 9 (45%)  6 (30%)  5 (25%)  0 (0%) |
| IGHV mutational status (n, %)  *Unmutated*  *Mutated* | 14 (70%)  6 (30%) | 0 (0%)  20 (100%) |

**Supplementary Table 7.** Baseline characteristics of the validation dataset published by Knisbacher *et al*.(3)

|  | **Total (n = 556)** |
| --- | --- |
| Gender (n, %)  *Male*  *Female* | 370 (67%)  186 (33%) |
| Age (median, IQR) | 63 (54-70) |
| RAI stage at sampling (n, %)  *0*  *I*  *II*  *III*  *IV*  *missing* | 10 (2%)  32 (6%)  63 (11%)  14 (3%)  48 (9%)  389 (69%) |
| IGHV mutational status (n, %)  *Unmutated*  *Mutated* | 252 (45%)  304 (55%) |
| IGLV3-21^R110^ (n, %)  *Present* | 50 (9%) |
| Epitype (n, %)  *n-CLL*  *i-CLL*  *m-CLL*  *unclassified*  *missing* | 211 (38%)  79 (14%)  216 (39%)  7 (1%)  43 (8%) |
| Cytogenetic aberrations (n, %)  *del(13q14.13)*  *del(13q14.2)*  *del(13q14.3)*  *del(11q22)*  *del(17p)*  *trisomy 12* | 105 (19%)  282 (51%)  156 (28%)  95 (17%)  23 (4%)  70 (13%) |
| TP53 mutation (n, %)  *Present* | 37 (7%) |

**Supplementary Table 8.** Cox proportional hazard models for failure-free survival generated using the dataset of Knisbacher *et al*.(3)

| **Failure-free survival** | **HR [±SE]** | **P-value** |
| --- | --- | --- |
| THEMIS2 gene expression  *per SD increase* | 2.03 [±0.24] | **0.003** |
| IGHV mutational status  *M-CLL*  *U-CLL* | -  2.84 [±0.12] | **-**  **<0.0001** |
| *TP53* aberrations  *none*  *any* | -  1.82 [±0.17] | **-**  **0.01** |
| IGLV3-21^R110^  *absent*  *present* | -  1.57 [±0.18] | **-**  **0.0004** |

**Supplementary Table 9.** Cox proportional hazard models for overall survival generated using the dataset of Knisbacher *et al*.(3)

| **Overall survival** | **HR [±SE]** | **P-value** |
| --- | --- | --- |
| THEMIS2 gene expression  *per SD increase* | 1.44 [±0.33] | 0.3 |
| IGHV mutational status  *M-CLL*  *U-CLL* | -  2.72 [±0.17] | **-**  **<0.0001** |
| *TP53* aberrations  *none*  *any* | -  1.42 [±0.27] | -  0.6 |
| IGLV3-21^R110^  *absent*  *present* | -  0.87 [±0.29] | -  0.6 |

**Supplementary Figure Legends**

**Supplementary Figure 1. Protein characterization quality control**

Barchart representing the percentage of missing or deviant protein expression values per sample. Samples with ≥40% missingness or deviancy were excluded from downstream analyses.

**Supplementary Figure 2. PCA and top 500 differentially expressed proteins**

A) Principal component analysis plot clustering patients, as represented by dots, by intracellular protein expression. The X- and Y-axis represent PCA1 and PCA2, respectively.

B) Heatmap and dendrogram generated by unsupervised hierarchical clustering, illustrating the relative abundance of the top 500 most differentially expressed proteins. Each row represents the expression of a protein, whereas each column represents a patient. Patient characteristics are represented by the ribbons in the top. The red-to-blue color scale indicates the Z-score.

Abbreviations: PCA; principal component analysis.

**Supplementary Figure 3.**

A) Dotplot demonstrating the normalized relative intracellular abundance of MARCKS, stratified per IGHV mutational status. Fold-change values were normalized to the average abundance of M-CLL samples. Statistical significance was evaluated using a *t-*test.

B) Dotplot demonstrating the normalized relative intracellular abundance of THEMIS2, stratified per light chain genotype. Fold-change values were normalized to the average abundance of control samples. Statistical significance was evaluated using a *t-*test.

C) Kaplan-Meier survival plot, estimating the proportion of failure-free patients over time, stratified by above or below average intracellular levels of THEMIS2 protein, quantified by mass spectrometry. Asterisks indicate right-censoring. Statistical significance was evaluated using a log-rank test.

D) Dot- and lineplots indicating the relative abundance of THEMIS2 protein expression in paired samples. The interval between the index sample and the repeated sample varied from 6-12 months. The blue line indicates the mean protein abundance. Correlation was evaluated using a Pearson’s test.

Abbreviations: CLL; chronic lymphocytic leukemia, IGHV; immunoglobulin heavy-chain variable, M-CLL; CLL with mutated IGHV, U-CLL; CLL with unmutated IGHV.

**References**

1. Therneau T. A Package for Survival Analysis in R. R package version 3.4-0.2022. Available from: https://cran.r-project.org/package=survival

2. Condoluci A, Di Bergamo LT, Langerbeins P, Hoechstetter MA, Herling CD, De Paoli L, et al. International prognostic score for asymptomatic early-stage chronic lymphocytic leukemia. Blood. 2020;135(21):1859–69.

3. Knisbacher BA, Lin Z, Hahn CK, Nadeu F, Duran-Ferrer M, Stevenson KE, et al. Molecular map of chronic lymphocytic leukemia and its impact on outcome. Nat Genet. 2022;54(November):1664–74.
